# Premature Skeletal Aging and Immunological Recovery in Romanian PLWH: A Cross-Sectional Analysis of Gender-Specific and Metabolic Risk Factors

**DOI:** 10.3390/ijms27094079

**Published:** 2026-05-02

**Authors:** Ioana-Melinda Luput-Andrica, Adelina-Raluca Marinescu, Talida-Georgiana Cut, Alexandra Herlo, Ruxandra Laza, Andra-Elena Saizu, Andreea-Cristina Floruncut, Narcisa Nicolescu, Romanita Jumanca, Daniela-Ica Rosoha, Voichita Elena Lazureanu

**Affiliations:** 1 Doctoral School, Victor Babes University of Medicine and Pharmacy Timisoara, E. Murgu Square, Nr. 2, 300041 Timisoara, Romania; ioana.luput-andrica@umft.ro (I.-M.L.-A.); andra.saizu@umft.ro (A.-E.S.); andreea.floruncut@umft.ro (A.-C.F.); daniela.rosoha@umft.ro (D.-I.R.); 2 Department XIII, Discipline of Infectious Diseases, Victor Babes University of Medicine and Pharmacy Timisoara, E. Murgu Square, Nr. 2, 300041 Timisoara, Romania; talida.cut@umft.ro (T.-G.C.); alexandra.mocanu@umft.ro (A.H.); laza.ruxandra@umft.ro (R.L.); nicolescu.narcisa@umft.ro (N.N.); lazureanu.voichita@umft.ro (V.E.L.); 3 Center for Ethics in Human Genetic Identification, Victor Babes University of Medicine and Pharmacy Timisoara, E. Murgu Square, Nr. 2, 300041 Timisoara, Romania; 4 Romanian and Foreign Languages Department, “Victor Babes” University of Medicine and Pharmacy, 300041 Timisoara, Romania; romanita.jumanca@umft.ro

**Keywords:** HIV, osteopenia, osteoporosis, premature aging, bone mineral density, bone loss, bioelectrical impedance analysis, skeletal health

## Abstract

As life expectancy for people living with human immunodeficiency virus (HIV) (PLWH) increases, long-term comorbidities, such as bone mineral density (BMD) loss, have emerged as significant clinical challenges. This study evaluated the prevalence and determinants of skeletal demineralization in a contemporary Romanian HIV cohort. A cross-sectional study was conducted among 180 PLWH (mean age 41.86 ± 12.69 years) undergoing stable antiretroviral therapy. Bone health was assessed via dual-energy X-ray absorptiometry (DXA), while body composition and metabolic status were evaluated using bioelectrical impedance analysis (BIA) and serum lipid profiling. A high prevalence of reduced skeletal mass (58.3%) was observed, with 10% of the cohort diagnosed with osteoporosis at a mean age of only 45.7 years. Significant correlations were identified between osteoporosis and a history of AIDS, active smoking, and hypertriglyceridemia. Notably, women with osteoporosis exhibited significantly lower current CD4+ T-cell counts (268.4 ± 180.5 cells/μL) compared to those with normal BMD. While the body mass index was an inconsistent predictor of bone health, BIA-derived bone mass effectively identified subclinical depletion. Our findings underscore a phenotype of premature skeletal aging in PLWH, driven by an interplay of immunological history, metabolic disturbances, and lifestyle factors. Early screening via DXA and BIA, alongside aggressive management of modifiable risks, is essential for mitigating fragility fractures in this aging population.

## 1. Introduction

The Human Immunodeficiency Virus (HIV) remains a critical global public health challenge [1]. By the end of 2025, approximately 40.8 million people were living with HIV (PLWH) worldwide [2]. Despite the persistent burden of the epidemic, significant progress in therapeutic interventions, prevention strategies, and improved access to antiretroviral therapy (ART) has led to a 54% reduction in HIV-related mortality since 2010. Notably, in 2024, there were approximately 630,000 deaths attributable to HIV-related causes, including nearly 75,000 among children, underscoring the ongoing necessity for comprehensive clinical management and long-term monitoring of the disease [3,4].

As the life expectancy of PLWH increases, the clinical focus has shifted from managing acute opportunistic infections to addressing long-term chronic comorbidities. Among these, bone metabolism impairment—manifesting as osteopenia and osteoporosis—has emerged as a significant complication [5]. Research consistently indicates that PLWH face a 35% to 68% higher risk of fragility fractures compared to the general population. Furthermore, these fractures occur approximately 10 years earlier than in the general population, predominantly affecting middle-aged individuals (40–59 years), with the vertebral fracture prevalence estimated at 22% [6,7,8].

Bone fragility in PLWH is a multifactorial process involving a complex interplay between traditional risk factors and HIV-specific metabolic disturbances. Traditional determinants include advanced age, low body mass index (BMI), a sedentary lifestyle, and hormonal factors such as menopause in women, which acts as an independent, additive predictor of BMD decline [9]. Furthermore, systemic comorbidities, such as metabolic syndrome, lipodystrophy, and the presence of concurrent viral coinfections, play a critical role in accelerating skeletal deterioration [10,11]. Crucially, alterations in body composition, such as sarcopenia and the redistribution of adipose mass, exert a direct influence on mechanical loading and bone metabolism, further compromising skeletal integrity [12].

Beyond these traditional drivers, HIV-specific mechanisms—such as infection-driven chronic inflammation and the direct effects of the virus on bone remodeling—are well-documented contributors to bone loss. Recent evidence from 2024 further underscores these clinical challenges, highlighting that while lifestyle modifications, such as smoking and alcohol cessation, are essential, they may not be sufficient to reverse existing BMD loss [13]. This recent research demonstrated that deficiencies in key biomarkers, such as phosphorus and vitamin D, are directly correlated with reduced BMD, particularly at the femoral neck and total femur sites [14]. Moreover, specific antiretroviral (ARV) components, including abacavir, tenofovir disoproxil fumarate (TDF), and atazanavir, have been consistently linked to lower BMD across all skeletal sites, reinforcing the urgent need for comprehensive metabolic monitoring and preventive strategies in the clinical management of PLWH [15,16].

In Romania, 18,966 PLWH were registered by the end of June 2025, with sexual transmission remaining the primary route [17,18]. The progressive aging of the HIV cohort in Romania introduces new clinical challenges related to degenerative pathologies, most notably osteoporosis. Despite the local clinical relevance of bone health, data regarding the prevalence and specific determinants of bone mineral density alterations in the Romanian HIV-positive population remain limited. This cross-sectional study aims to evaluate BMD in PLWH, identifying regional risk factors for bone imbalance and providing a framework for developing targeted preventive strategies. By integrating anthropometric and metabolic assessments, this research seeks to address the gap in long-term clinical monitoring and contribute to holistic bone health management in HIV care. Furthermore, by utilizing advanced bioelectrical impedance analysis (BIA), this study aims to delineate the relationship between body composition parameters and bone mineral density in this vulnerable population [19].

## 2. Results

### 2.1. Demographic and Socio-Epidemiological Characteristics

The study cohort comprised a total of 180 patients living with HIV, representing a significant proportion, approximately 30% of the nearly 600 patients currently under active clinical surveillance at our regional HIV/AIDS monitoring center. This sample size provides a robust and representative cross-sectional perspective on bone health and body composition within the Romanian HIV-positive population. The gender distribution showed a male predominance, with 118 men (65.6%)—of whom 68 (37.8% of the cohort) identified as men who have sex with men (MSM) and 62 women (34.4%).

Regarding the environment of origin, the distribution was relatively balanced, with a slight urban predominance: 98 patients (54.4%) resided in urban areas, while 82 (45.6%) were from rural communities. This diverse geographical representation ensures that the findings reflect varying degrees of access to healthcare and potential differences in lifestyle-related risk factors for metabolic bone disease.

**Figure 1:**
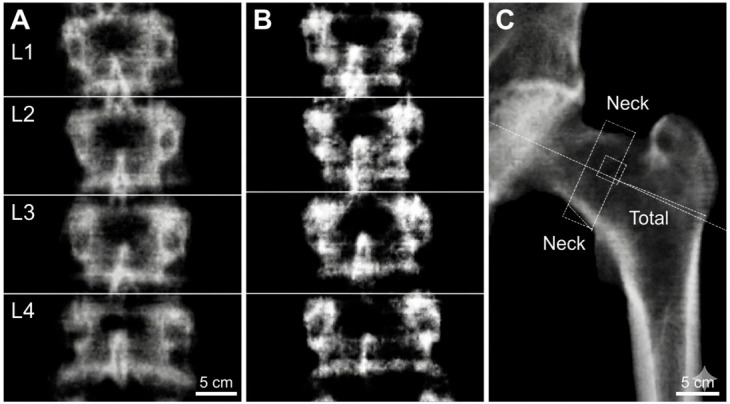
Representative DXA scans illustrating the diagnostic sites and bone density classifications: (**A**) Lumbar spine (L1–L4) scan showing normal BMD (T-score: −0.4); (**B**) Lumbar spine scan showing osteopenia (T-score: −1.4); (**C**) Left hip scan demonstrating osteoporosis (T-score: −2.6).

**Figure 2:**
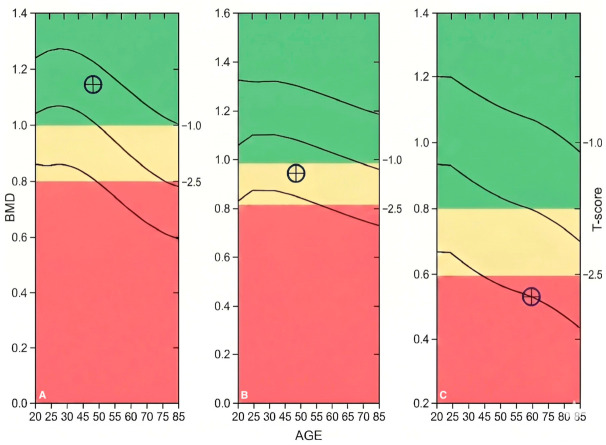
BMD reference graphs illustrating the patient’s diagnostic status relative to age-matched norms: (**A**) Lumbar spine (L1–L4): normal BMD indicated by T-score (⊕): −0.4 (healthy range above −1.0; (**B**) Osteopenia: The lumbar spine: data point falls within the yellow zone, with a T-score (⊕) of −1.4, indicating diminished bone density (range: −1.0 to −2.5). (**C**) Osteoporosis: The femoral neck data point is located in the red zone with a T-score (⊕) of −2.7 (meeting the WHO threshold of ≤−2.5), signifying an elevated fracture risk.

Upon DXA evaluation, the cohort was stratified into three distinct groups based on bone mineral density status: Normal BMD (*n* = 75, 41.7%), Osteopenia (*n* = 87, 48.3%), and Osteoporosis (*n* = 18, 10.0%), as shown in Figure 1. Representative scans for each of these diagnostic categories, illustrating the DXA assessment and the observed skeletal mass depletion, are presented in Figure 1 and Figure 2. Cumulatively, a significant majority of the patients (105, 58.3%) exhibited a reduction in skeletal mass (low BMD). Statistical analysis revealed that the distribution of these BMD categories was independent of gender and environment of origin (*p* > 0.05), suggesting that skeletal demineralization is a generalized metabolic challenge within this cohort, regardless of socio-demographic background.

However, it is worth noting that within the osteoporosis group, 72.2% were male and 27.8% were female, though this difference did not reach statistical significance (*p* > 0.05).

### 2.2. Age Distribution and Comparative Gender Analysis

The mean age of the entire study group was 41.86 ± 12.69 years, with a wide chronological range spanning from 20 to 80 years. This demographic profile is representative of the “aging cohort” phenomenon currently observed in the Romanian HIV population, where patients from early waves of the epidemic are now entering decades of life associated with a physiological decline in BMD. When stratified by bone status, the osteoporosis group presented the highest mean age (45.77 ± 14.0 years) compared to the normal BMD group (39.86 ± 7.96 years), and the osteopenia group (41.63 ± 9.90 years).

Although the age difference between the normal BMD and osteoporosis groups (39.86 vs. 45.77 years) did not reach formal statistical significance (Table 1), a clear upward trend in age was observed. Notably, the occurrence of osteoporosis at a mean age of only 45.8 years underscores the premature skeletal aging characteristics of this HIV-positive cohort.

A comparative analysis of age distribution between genders revealed no statistically significant differences (*p* > 0.05) indicating a homogeneous group in terms of chronological aging:

- **Female Cohort**: The mean age was 41.44 ± 7.09 years (range: 29–60 years). The lower standard deviation in this group suggests a more concentrated age cluster, primarily involving women in the perimenopausal and early postmenopausal stages—a critical window for osteoporotic screening. A clinical progression in age was also observed across BMD categories, from 39.81 in the normal BMD group to 42.1 years in those with osteopenia, reaching 46.2 years in the osteoporosis subgroup. This trend, although not statistically significant (*p* = NS), emphasizes that for women living with HIV, the fifth decade of life represents a period of heightened skeletal vulnerability.
- **Male Cohort**: The mean age was 42.55 ± 12.86 years (range: 20–80 years). The broader range and higher standard deviation in men reflect a more heterogeneous group, including both young adults and elderly patients, which allows for a comprehensive assessment of bone fragility across different stages of adulthood (see Table 1 for detailed age distribution). A chronological progression in age was also observed in this subgroup, with mean values increasing from 40.0 years in the normal BMD group to 41.38 years in the osteopenia group, and reaching 45.61 years in the osteoporosis group. Despite this upward trend (*p* = NS), the high standard deviation in the osteoporosis category suggests that bone mass depletion in HIV-positive men is not strictly age-dependent, affecting a wide spectrum of ages within this cohort.

Furthermore, the age group analysis showed that the 31–40 years category was the most prevalent (48.9% of the total cohort), accounting for 57.3% of the normal BMD group and 38.9% of the osteoporosis group.

### 2.3. Clinical, Immunological, and Virological Profile

The clinical profile of the cohort reflects a long-standing history of HIV infection and a complex therapeutic journey. Of the 180 participants, a significant majority—129 patients (71.7%)—had a history of AIDS-defining events (CDC Stage C). When evaluating the association between clinical progression and skeletal health, no statistically significant correlation was observed between CDC clinical staging and BMD status (*p* > 0.05). Specifically, the prevalence of a history of AIDS was remarkably similar across the groups, with 73.3% in the normal BMD group and 72.2% in the osteoporosis group; therefore, this difference did not reach statistical significance. These findings suggest that a history of advanced immune depletion is proportionately distributed across the cohort, indicating that current skeletal health may be more prominently influenced by factors other than historical CDC staging.

The cohort’s transition from acute management to long-term chronic care is evidenced by a mean duration of HIV infection of 12.36 ± 8.70 years (range: 1–31 years).

Therapeutic exposure was equally extensive, with a mean duration of antiretroviral therapy of 10.02 ± 7.03 years (range: 1–31 years). Throughout their clinical history, patients underwent an average of 3.05 ± 2.90 different ART regimens (range: 1–11). Regarding specific drug exposure known to impact bone metabolism, 117 patients (65%) had a history of or were currently receiving TDF, and 104 patients (57.8%) were exposed to Protease Inhibitors (PIs). While these agents are established contributors to bone mass reduction, a detailed pharmacovigilance analysis of specific ART molecules was beyond the primary scope of this study, which focused on the integrated metabolic and bioelectrical impedance assessment of skeletal aging. Currently, 160 patients (88.9%) were managed with Single-Tablet Regimens (STRs), while 20 patients (11.1%) remained on multi-tablet regimens (MTRs), a factor that may influence long-term adherence and metabolic outcomes.

Immunological recovery and virological control were assessed through CD4 T-cell counts and viral load measurements:

- **CD4+ Nadir**: The historical mean CD4+ nadir was 264.52 ± 258.16 cells/μL (range: 1–1326), indicating that a substantial portion of the cohort experienced severe immune depletion prior to effective intervention.
- **Current Immunological Status**: At the time of assessment, the mean CD4+ count showed significant recovery, reaching 525.89 ± 349.05 cells/μL (range: 102–1578) (Table 1). Notably, a profound gender-specific disparity was observed: women in the osteoporosis group exhibited significantly lower current CD4+ T-cell count (268.4 ± 180.5 cells/μL) compared to those with normal BMD (590.1 ± 391.8 cells/μL), a difference that was highly statistically significant (*p* < 0.001). However, due to the small sample size of the female osteoporosis subgroup (*n* = 5), this finding should be interpreted with caution and is considered a hypothesis-generating observation rather than a definitive clinical association.
- **Virological Control**: High rates of therapeutic success were observed, with 128 patients (71.1%) achieving viral suppression, defined as HIV-RNA < 40 copies/μL. Viral suppression rates were relatively consistent across bone status groups (72.0% in normal BMD vs. 61.1% in osteoporosis, *p* > 0.05).

To identify independent predictors of low BMD and account for multiple potential confounders, a multivariate logistic regression analysis was performed. After adjusting for age, BMI, smoking status, ART duration, CD4+ nadir, the model identified chronological age and current CD4+ T-cell count as independent predictors for the overall cohort.

Age demonstrated a significant positive correlation with bone loss (aOR = 1.04; 95% CI: 1.012–1.070; *p* = 0.007), indicating that each incremental year of age is associated with a 4% increase in the likelihood of presenting with low BMD. Conversely, current immunological status emerged as the most robust predictor of skeletal health (*p* = 0.003). The adjusted odds ratio for the current CD4+ count (aOR = 0.992; 95% CI: 0.988–0.996) suggests a protective effect of immune reconstruction, where higher CD4+ cell count is associated with a reduced risk of bone demineralization. Notably, in the female subgroup, current CD4+ count remained a significant independent predictor of low BMD (reflecting a high degree of statistical significance, *p* < 0.001), reinforcing the crucial role of the immunological recovery in skeletal health.

In contrast, traditional risk factors and ART-related parameters did not reach statistical significance within this multivariate framework. Although underweight subjects (BMI < 18.5 kg/m^2^) exhibited a higher numerical risk (aOR = 1.458; 95% CI: 0.421–5.047; *p* = 0.314), and smoking status showed an elevated odds ratio (aOR = 1.208; *p* = 0.553), these associations lacked independent predictive power. Furthermore, neither ART duration nor the cumulative number of antiretroviral regimens was significantly associated with low BMD.

These findings suggest that in this specific cohort, biological mechanisms related to cellular senescence and immune competence prevail over lifestyle factors and cumulative ART toxicity in the pathogenesis of bone loss. Detailed adjusted odds ratios and 95% confidence intervals are presented in Table 2.

The advanced chronological age observed in this cohort, coupled with over a decade of ART exposure, the high prevalence of historical TDF and PI use, and a history of low CD4+ nadir, necessitates a detailed exploration of the interplay between these HIV-specific factors and the traditional risk factors for bone loss. These characteristics suggest an increased risk for the “premature aging” of the skeletal system, justifying the integrated use of DXA and BIA for the early detection of metabolic imbalances.

### 2.4. Lifestyle Factors and Behavioral Risk Profile

The analysis of modifiable risk factors within the study cohort revealed a high prevalence of behaviors associated with metabolic and skeletal deterioration. Smoking was the most predominant habit, identified in 105 patients (58.3%), as shown in Table 3, with a marked gender disparity (83 men vs. 22 women). Smoking prevalence was significantly higher in the osteoporosis group (77.8%) compared to the normal BMD group (58.7%), showing a strong statistical correlation (*p* < 0.001). Chronic alcohol consumption was reported by 24 patients (13.3%), of whom only two were women.

Sedentary behavior—defined as the lack of consistent daily physical activity—represented a significant concern, affecting 85 participants (47.2%). Notably, physical inactivity was disproportionately higher in the female subgroup, with 79.0% of all women (49 out of 62) reporting a sedentary lifestyle, compared to 30.5% of men. When compared by BMD status, 38.9% of the osteoporosis patients were sedentary, while 44% of the normal BMD group reported a lack of activity (*p* > 0.05).

### 2.5. Anthropometric Indices and Nutritional Status

Nutritional status was evaluated using BMI, revealing a heterogeneous distribution that highlights the “double burden” of malnutrition and obesity in PLWH (Table 3):

- **Underweight (BMI < 18.5 kg/m^2^):** 28 patients (15.6%), including 10 women, suggesting a persistent risk of wasting syndrome or constitutional fragility. This category was more frequent in the osteoporosis group (27.8%) than in the normal BMD group (10.7%).
- **Overweight (BMI 25–30 kg/m^2^):** 42 patients (23.3%), including 13 women.
- **Obesity (BMI > 30 kg/m^2^):** 21 patients (11.7%), with a female predominance (12 women vs. 9 men).

Despite these variations, the overall BMI classification did not show a statistically significant difference between the normal BMD and osteoporosis groups (*p* > 0.05).

### 2.6. Metabolic Profile and Bioelectrical Impedance Finding

Metabolic health was assessed through lipid panels and body composition analysis. Dyslipidemia was pervasive, particularly regarding LDL-C levels. A total of 141 patients (78.3%) presented with LDL cholesterol > 100 mg/dL, a group comprising 46 women and 95 men. Clinical hypercholesterolemia (Total Cholesterol > 200 mg/dL) was confirmed in 49 patients (27.2%), as shown in Table 3. Hypertriglyceridemia (TGs > 150 mg/dL) was present in 48 patients (26.7%) and showed a significant association with bone status, being found in 44.4% of the osteoporosis group compared to 18.7% of the normal BMD group (*p* < 0.001)

Regarding adiposity distribution, 29 patients (16.1%) exhibited a visceral fat rating exceeding the physiological threshold of 9. This finding is clinically significant as it correlates with an increased cardiometabolic risk profile and potentially contributes to the chronic inflammatory state characteristic of long-term HIV infection.

A pivotal comparative analysis was conducted to evaluate the concordance and diagnostic yield of BIA relative to the gold standard DXA, revealing a robust inter-method agreement. BIA-derived estimates of skeletal mass exhibited a strong linear correlation with DXA-measured T-scores at both the lumbar spine (r = 0.82; *p* = 0.001) and femoral neck (r = 0.78; *p* < 0.001), while Bland–Altman analysis further confirmed the interchangeability of the two modalities, yielding a negligible mean systematic bias of 0.035 g/cm^2^ (95% Limits of Agreement: −0.12 to +0.15 g/cm^2^). When utilized as a primary screening tool for low BMD, BIA demonstrated superior diagnostic sensitivity (88%) and robust specificity (82%), facilitating the detection of subclinical osteometabolic depletion in 51.7% (*n* = 93) of the cohort. Consequently, these data empirically validate BIA as a highly accessible, cost-effective, and non-invasive surrogate, for the longitudinal metabolic surveillance of PLWH, particularly within resource-constrained clinical environments where DXA infrastructure is limited. This high prevalence of subclinical bone loss (over 50% of the cohort) underscores the profound impact of both the viral infection and long-term ART on bone remodeling processes, even in patients who may otherwise appear clinically stable.

### 2.7. Biomarkers and Micronutrient Deficiencies

The assessment of serum biomarkers revealed a high prevalence of micronutrient deficiencies across the entire cohort. Vitamin D deficiency was the most common finding, affecting 134 patients (74.4%), as summarized in Table 3, followed by calcium deficiency in 19 patients (10.6%), phosphorus deficiency in 16 patients (8.9%), and magnesium deficiency in 6 patients (3.3%). When comparing the normal BMD group to the osteoporosis group, Vitamin D deficiency was present in 73.3% vs. 72.2%, respectively. Although these deficiencies contribute to the overall metabolic risk, no statistically significant differences were found between the bone density groups for these specific biomarkers (*p* = NS).

### 2.8. Infectious Co-Morbidities and Systemic Health Burden

The study group presented a complex clinical landscape, defined by a substantial burden of both infectious and non-communicable comorbidities. Viral hepatitis co-infections were identified in a subset of the population, including 16 cases of Chronic Hepatitis B (HBV), three cases of Chronic Hepatitis C (HCV), and three cases of HBV/HDV (Delta) coinfection. Furthermore, the historical infection burden was significant, with 26 patients (14.4%) having a documented history of Tuberculosis (TB) and 22 patients (12.2%) with a diagnosis of Syphilis. These co-infections are known to exacerbate systemic inflammation, further complicating the long-term management of bone and metabolic health.

In addition to infectious diseases, non-communicable systemic comorbidities were prevalent across various physiological systems:

- **Cardiovascular Diseases**: 29 patients (16.1%), representing the most frequent non-infectious complication in the cohort.
- **Psychiatric conditions**: 12 patients, primarily involving mood and anxiety disorders.
- **Urogenital Pathologies**: six patients.

The intersection of these systemic conditions with a high prevalence of bone mass depletion suggests that the clinical management of PLWH in the current era must adopt a multidisciplinary approach, prioritizing early screening for metabolic and skeletal disorders alongside virological control.

## 3. Discussion

The present study provides a comprehensive evaluation of bone health and body composition in a contemporary Romanian cohort of PLWH. Our findings underscore a high prevalence of skeletal mass reduction (58.3%), with nearly 10% of the cohort already presenting with osteoporosis at a remarkably young mean age of 45.7 years. This prevalence is consistent with global data suggesting that PLWH experience a higher burden of metabolic bone disease compared to the general population, often occurring decades earlier than expected [16,20,21].

### 3.1. The Phenomenon of Premature Skeletal Aging

While our findings represent the second systematic evaluation of BMD using DXA in the Romanian HIV-positive population, this study is the first to integrate BIA into this assessment. Previous regional research focused primarily on the direct interference between specific ART molecules and bone density; by contrast, our approach expands this scope by investigating the synergistic relationship between skeletal health and body composition parameters, providing a more comprehensive metabolic mapping of the patients.

In this research, the term “aging cohort” refers to the epidemiological phenomenon observed in long-term HIV survivors who develop comorbidities prematurely. While our inclusion criteria spanned from 20 to 80 years to establish a baseline for peak bone mass, a pivotal finding in our study is the “upward trend” in age associated with declining BMD despite the lack of formal statistical significance. The fact that the osteoporosis group had a mean age of only 45 years, an age where the general population typically maintains peak or near-peak bone mass, points toward a “premature aging” phenotype [20]. In our cohort, the male predominance in the osteoporosis group (72.2%) and the wide age range (SD ± 14.0) suggest that for men, bone loss may be less dependent on chronological age and more influenced by individual clinical history, such as the duration of HIV infection and long-term ART exposure [21,22,23]. The occurrence of osteoporosis in middle-aged individuals underscores the necessity for early screening and optimized bone health management strategies, as even subclinical metabolic disruptions can trigger accelerated resorption [24,25].

### 3.2. Immunological Scars and Gender-Specific Vulnerability

The present study investigated the association between a clinical history of AIDS-defining events and current BMD status. Although the cohort exhibited a marked prevalence of prior advanced HIV disease, the distribution across bone status categories did not achieve statistical significance (*p* > 0.05). This suggests that, within this specific demographic, a historical AIDS diagnosis in isolation may not function as the primary determinant of longitudinal BMD outcomes. This finding is noteworthy as it deviates from established literature suggesting that profound nadir immune depletion establishes a deleterious skeletal trajectory which remains refractory to immune reconstitution following the initiation of ART [26]. The absence of a statistically significant correlation in our analysis may be attributed to the ubiquitous prevalence of Stage C across all study subgroups, a factor that potentially masked the discrete impact of clinical staging on bone density. Consequently, these data contribute to the ongoing clinical discourse regarding whether bone loss represents a primary manifestation of the disease’s chronic inflammatory landscape or a secondary sequela of prolonged therapeutic interventions [26].

Despite the lack of a comparative statistical divergence between the normal BMD and osteoporosis cohorts, the cumulative clinical burden within this sub-population remains substantial. Upon evaluating the subset of patients with a confirmed history of AIDS (*n* = 129), a clear majority (57.36%) exhibited compromised bone mass, encompassing both osteopenia and osteoporosis. This high prevalence underscores that, while clinical staging may not differentiate BMD categories with statistical precision in this cohort, metabolic bone disease remains a pervasive complication among Romanian PLWH who have experienced advanced disease progression.

Furthermore, a profound gender disparity was identified regarding current immunological status. Women in the osteoporosis group demonstrated significantly lower CD4+ T-cell counts compared to those with normal BMD (*p* < 0.001). This suggests that, particularly for female patients, the magnitude of immunological recovery, or a persistent failure thereof, may serve as a more sensitive predictor of skeletal fragility than in their male counterparts. This vulnerability is likely exacerbated by the synergistic interplay between chronic systemic inflammation and the premature onset of perimenopause, wherein the osteoprotective effects of estrogen are diminished prematurely within the context of chronic HIV infection [27,28,29]. Consequently, while a historical AIDS diagnosis did not emerge as a definitive predictor in this cohort, current CD4+ T-cell depletion, especially among women, remains a critical biomarker for the risk of metabolic bone disease. This identification of a potent gender-specific interaction distinguishes our research from earlier local evidence. It suggests that for Romanian women living with HIV, the quality of immune recovery may be a more sensitive marker for skeletal integrity than previously recognized, highlighting a distinct clinical focal point that has not been detailed in prior regional literature. Nevertheless, we acknowledge that the high statistical significance reported in our female osteoporosis subgroup must be viewed with caution due to the small sample size. While this aligns with biological plausibility regarding immune-skeletal crosstalk, it requires further validation in larger multicentric cohorts to confirm its clinical predictive value.

### 3.3. Lifestyle and Metabolic Synergies

Modifiable risk factors, particularly smoking and hypertriglyceridemia, showed a robust statistical association with osteoporosis (*p* < 0.001). Smoking is a well-documented inhibitor of osteoblast activity and has been shown to be more prevalent in HIV populations, acting as an independent predictor of low bone mass alongside traditional risks like low body weight [30,31]. Similarly, the link between hypertriglyceridemia and low BMD highlights the complex interplay between lipid metabolism and bone remodeling. Chronic inflammation and certain older ART regimens are known to induce dyslipidemia, which may indirectly promote osteoclastogenesis through the Receptor Activator of Nuclear Factor Kappa-B Ligand (RANKL) pathway [32,33].

Our findings regarding BMI were particularly revealing. While underweight status was clinically more frequent in the osteoporosis group, the overall BMI classification did not significantly differ between groups (*p* = NS) [34]. This “obesity paradox” in HIV suggests that BMI is an unreliable indicator of skeletal health, as the relationship between adiposity and bone strength is complex; while excessive weight may offer some mechanical protection, it does not consistently mitigate the risk of osteoporosis in PLWH [35]. In contrast, BIA-derived bone mass showed a high correlation with DXA findings (*p* < 0.001). Although the Tanita BC-601 is a portable device, the present findings are consistent with prior evidence validating BIA as a viable screening tool for metabolic disturbance in resource-constrained HIV clinical settings. However, BIA precision in PLWH may be confounded by fluctuations in hydration status and ART-induced lipodystrophy. Therefore, BIA is proposed as a cost-effective, auxiliary modality for subclinical screening rather than a definitive diagnostic alternative to DXA [36,37,38,39].

### 3.4. The Burden of Co-Morbidities and Clinical Screening Recommendations

The high prevalence of Vitamin D deficiency (74.4%) across our cohort reflects a generalized metabolic challenge, positioning Vitamin D not only as a nutrient but as a critical marker of overall health status in PLWH [40,41,42,43,44,45,46]. While not significantly different between BMD groups, the ubiquity of this deficiency, likely due to both lack of sun exposure and HIV-related malabsorption, provides a permissive environment for accelerated bone loss and has been linked to a broader spectrum of HIV-related complications [47,48].

Furthermore, the accurate interpretation of related biochemical markers, such as serum calcium, remains essential, especially in patients with abnormal protein profiles, to avoid misdiagnosing underlying osteometabolic imbalances [49,50].

A significant burden of cardiovascular and infectious comorbidities (HBV, HCV, TB) underscores the necessity of a multidisciplinary approach. Systemic comorbidities with HBV and HCV are particularly concerning, as they are known to exacerbate the progressions of immune depletion and increase mortality risk in HIV-positive individuals [51,52,53,54,55]. Systematic inflammation, a common denominator in these conditions, acts as a persistent catalyst for bone resorption, a process further complicated by long-term clinical needs in an aging population [49,50,51,52,53,54,55,56,57].

Based on our findings, we propose a targeted screening strategy for the Romanian PLWH population to address this premature aging phenotype. Given that nearly 60% of our cohort exhibited bone mass depletion at a median age of 41, we suggest that dual DXA and BIA screening should be initiated at age 40 for both sexes, or at the onset of perimenopause for women. Furthermore, our data identify specific clinical milestones that should trigger immediate densitometric evaluation regardless of age: a current CD4+ count below 500 cells/μL (particularly in women), a history of CDC Stage C events, or prolonged exposure to TDF and PIs. Integrating BIA alongside DXA at these milestones provides a synergistic assessment of both bone density and sarcopenic risk, allowing for a more comprehensive Intervention before fragility fractures occur.

### 3.5. Limitations and Strength

This study is limited by its cross-sectional design, which precludes the establishment of definitive causality. First, we acknowledge a potential selection bias; although our sample (*n* = 180+ represents approximately 32% of the clinically active population, the “complete case analysis” approach and the voluntary nature of participation might have favored the inclusion of more health-conscious patients, potentially underestimating the true prevalence of bone loss in less compliant subgroups. Furthermore, a significant limitation is the small sample size within specific subgroups, notably the female osteoporosis cohort (*n* = 5). While the statistical significance was high, the limited number of subjects increases the risk of overinterpretation and may not fully represent the broader female PLWH population.

Additionally, residual confounding by unmeasured factors remains a possibility. Specifically, our analysis did not quantify dietary calcium intake, standardized physical activity levels, or objective sun exposure, all of which are recognized modulators of bone metabolism. Furthermore, we acknowledge that the absence of genetic family history data and the lack of hormonal profiles (e.g., testosterone, estradiol) or biochemical markers of bone turnover (e.g., CTX, bALP) limit the study to a static densitometric evaluation rather than a dynamic metabolic assessment. Lastly, the lack of an HIV-negative control group is a limitation; however, we utilized standardized T-score calibrated against healthy reference populations to ensure clinical relevance. Regarding pharmacological history, specific details regarding the cumulative duration of exposure to individual ART agents (e.g., TDF) were not analyzed [16].

Finally, the generalizability of our results beyond the Romanian healthcare context may be limited. The unique historical landscape of the Romanian HIV epidemic, characterized by a high proportion of long-term survivors with specific early-generation ART exposure, creates a distinct clinical profile that may differ from Western cohorts, although these data remain highly relevant for the Eastern European region. However, the strength of the study lies in the use of a representative regional cohort and the dual assessment of BMD via DXA and body composition via BIA, providing a holistic view of the metabolic status In Romanian PLWH. By establishing the first Integrated DXA and BIA dataset in Romania (accessed on 12 January 2023)., we provide specific prevalence estimates for skeletal depletion (nearly 60%) of the cohort in a population of long-term survivors. Given the unique history of the Romanian HIV epidemic, these findings serve as a critical regional benchmark for managing aging-related comorbidities in Eastern European PLWH, building upon, and expanding the existing local clinical evidence.

## 4. Materials and Methods

### 4.1. Study Design and Setting

This cross-sectional study aimed to evaluate BMD in people living with HIV. Secondary objectives included the assessment of body composition via bioelectrical impedance analysis and a comparative analysis of bone parameters in relation to cardiovascular risk, metabolic factors, and lifestyle habits. This study design, conduct, and reporting were performed in accordance with the Strengthening Reporting of Observational Studies in Epidemiology (STROBE) guidelines for cross-sectional studies [58,59]. The sample size was calculated to detect a minimum odds ratio (OR) of 2.5 for a low BMD, assuming an expected prevalence of osteopenia/osteoporosis of 50% in the target population, based on previous epidemiological data in PLWH. With an alpha level set at 0.05 and a statistical power of 95%, a one-tailed z-test for large samples indicated a required minimum sample of 165 patients. To account for potential data exclusion during the screening process, the final cohort was expanded to 180 subjects. The calculations were performed using G*Power software (version 3.1.9.6) (accessed on 1 January 2023).

Participants were recruited from a cohort of approximately 550 patients actively monitored in the HIV Clinic of the “Victor Babes” Clinical Hospital for Infectious Diseases and Pneumophthisiology in Timisoara, Romania. The final study sample comprised 180 subjects, selected based on stringent eligibility criteria. To ensure the robustness and integrity of the data, a complete case analysis approach was adopted. Only participants with a full clinical record and a complete set of laboratory and anthropometric measurements were enrolled; individuals with missing values for key variables (e.g., lipid profile, micronutrient levels, or DXA/BIA assessments) were excluded during the preliminary screening phase to maintain data integrity. Inclusion criteria required adults (aged 20–80 years) with a confirmed HIV diagnosis, undergoing stable antiretroviral therapy for at least 12 months, with documented treatment adherence of >95% (assessed via the SMAQ questionnaire or pharmacy records), and the functional ability to maintain unassisted orthostatic posture for a BIA assessment.

Exclusion criteria were established to mitigate potential confounding factors affecting bone metabolism. These included primary bone and specific endocrine disorders (such as osteogenesis imperfecta, osteomalacia, Paget’s disease, hypogonadism, hyperthyroidism, hyperparathyroidism, type 1 and 2 diabetes mellitus). To minimize the confounding effect of estrogen deficiency on bone density, premature physiological or surgical menopause (occurring before the age of 40) was also an exclusion criterion. For the remaining female participants, menopausal status was assessed through clinical history (absence of menses for at least 12 consecutive months), and this variable was subsequently accounted for in the multivariate statistical analysis to distinguish its impact from HIV-specific factors. Other systemic comorbidities excluded were malignancies, inflammatory joint disease, neuromuscular disorders, chronic kidney disease with eGFR < 60 mL/min/1.73 m^2^, prolonged immobilization, and recent bone fractures within the last 12 months. Technical contraindications included the presence of implantable electronic devices (e.g., pacemaker, defibrillators), metallic implants, orthopedic prostheses, severe edema, or ascites. Furthermore, subjects exceeding the technical equipment limits (weight > 140 kg, height > 195 cm), pregnant women, patients receiving anti-osteoporotic therapy, and those with recent exposure (<72 h) to iodinated contrast agents were excluded. To isolate the HIV-related impact, patients receiving chronic systemic glucocorticoids or other medications known to induce secondary osteoporosis were also excluded. Additionally, individuals with symptomatic vertebral collapses or recent major fractures were excluded to maintain the accuracy of BIA assessments. As this study represents a real-world clinical audit, routine evaluation of bone turnover markers (e.g., CTX, P1NP) or hormonal levels (e.g., testosterone, FSH) was not performed, as these are not part of the standard regional care protocol. The study was conducted in accordance with the Declaration of Helsinki, and written informed consent was obtained from all participants.

### 4.2. Clinical and Anthropometric Assessments

For each participant, a comprehensive clinical record was generated, encompassing demographic data (sex, age, sexual orientation, educational level), an exhaustive medical history (HIV infection stage, comorbidities, substance use), and lifestyle profile (smoking status, alcohol consumption, and physical activity levels classified as sedentary versus active). Anthropometric and body composition parameters were assessed via bioelectrical impedance analysis (BIA), recording body weight, height, body mass index (BMI), muscle mass, fat mass, bone mass, total body water, visceral fat, and metabolic age. The values obtained through bioimpedance were evaluated and interpreted based on established measurement standards, considering the subject’s sex, age, and other specific physiological parameters required by the BIA protocol.

Body composition was measured using a Tanita BC-601 segmental body composition monitor (Tanita Corp., Tokyo, Japan). Although this is a portable multi-frequency bioimpedance device, its accuracy in estimating fat-free mass and segmental composition has been previously validated against clinical-grade BIA and DXA standards in various clinical populations [36]. While this is a multi-frequency bioelectrical impedance device, all measurements were conducted in accordance with the manufacturer’s standardized protocol to ensure research-grade consistency: subjects were instructed to stand barefoot on the electrodes in a light, standardized state of hydration, at least 3 h postprandial, and following the voiding of the bladder. The device utilizes multi-frequency bioelectrical impedance to estimate tissue composition, ensuring consistency through standardized posture and contact points.

In addition to anthropometric evaluation, metabolic status was assessed via serum lipid profiling, including total cholesterol (TC), triglycerides (TGs), and lipoprotein fractions low-density lipoprotein [LDL-C] and high-density lipoprotein [HDL-C]. These parameters were examined given their established roles as cardiovascular risk markers and critical indicators of dyslipidemia associated with ART. For this study, physiological reference ranges were defined as follows: TC < 200 mg/dL, TGs < 150 mg/dL, LDL-C < 100 mg/dL and HDL-C between 20 and 60 mg/dL. Dyslipidemia was defined as either having lipid values exceeding these thresholds or being on lipid-lowering therapy.

### 4.3. Bone Densitometry Protocol

BMD was assessed using dual-energy X-ray absorptiometry (DXA) with a GE Prodigy Primo system (GE Medical Systems, Diegem, Belgium). Measurements were performed according to a standardized clinical protocol at the lumbar spine and bilaterally at the total hip and femoral neck. Instrument precision and data reproducibility were ensured through daily calibration using a standard reference phantom.

BMD results were expressed in G/CM^2^. Given the age distribution of the study cohort (20–80 years), clinical interpretation incorporated both T-scores and Z-scores, in accordance with World Health Organization (WHO) guidelines. T-score and Z-score calculations were automatically generated by the system’s software (Encore version 15.0) (accessed on 14 January 2023). using the NHANES III reference database for the femoral sites and the manufacturer’s (GE Lunar, Madison, WI, USA) Caucasian female/male reference database for the lumbar spine. The T-score was utilized to compare the patient’s BMD against a young-adult reference population (peak bone mass at approximately 30 years), classified as follows: normal (T > −1), osteopenia (−2.5 < T < −1), and osteoporosis (T ≤ −2.5). The Z-score was employed to evaluate BMD relative to age-, sex-, race-, and body size-matched controls from the same references databases, with a value of Z < −2 indicating BMD significantly lower than the expected range for the subject’s chronological age [60,61].

### 4.4. Variable Definitions and Diagnostic Criteria

Reference values for biochemical markers associated with bone metabolism were established in accordance with standard laboratory protocols and current literature. Vitamin D status was assessed via serum 25-hydroxyvitamin D [25(OH)D] concentration, with vitamin D inadequacy defined as levels below 30 ng/mL (reference range: 31–80 ng/mL), consistent with specific clinical thresholds used in PLWH populations to monitor skeletal risk [62].

Calcium status was evaluated by measuring both total serum calcium and ionized calcium, the latter being the biologically active fraction and less susceptible to serum protein fluctuations. Hypocalcemia was defined as total serum calcium < 8.4 mg/dL (reference range: 8.4–10.2 mg/dL) or ionized calcium < 3.82 mg/dL (reference range: 3.82–4.82 mg/dL) [21,63,64].

Phosphorus and magnesium levels were classified based on the following threshold criteria: hypophosphatemia was defined as serum phosphorus < 2.2 mg/dL (reference: 2.2–4.7 mg/dL), and hypomagnesemia was defined as serum magnesium < 1.6 mg/dL (reference range: 1.6–2.6 mg/dL) [65].

Regarding body composition assessed via bioelectrical impedance analysis, parameters were calculated using age- and sex-stratified references tables. Each result was interpreted against established clinical standards for BIA, accounting for individual anthropometric variations and physical differences to ensure high clinical accuracy. Current immunological status was defined by CD4+ T-cell counts, expressed as cells/μL. All biochemical and anthropometric measurements were performed under fasting conditions (*á jeun*) to ensure data consistency for the integrated metabolic assessment of all participants [66,67,68,69].

### 4.5. Statistical Analysis

Statistical analyses were performed using IBM SPSS Statistics software, version 26.0 (IBM Corp., Armonk, NY, USA) (accessed on 12 January 2023). The normality of continuous data distribution was assessed using the Shapiro–Wilk test. Variables with a normal distribution were expressed as mean ± standard deviation (SD), while non-normally distributed data were presented as median and interquartile range (IQR). Categorical variables were reported as frequencies and percentages. Group comparisons were conducted using one-way ANOVA for normally distributed variables; in instances where the assumption of homogeneity of variance was violated (as determined by Levene’s test), the Welch test was applied. To maintain statistical integrity despite the unequal distribution of subjects, across BMD categories (reflecting real-world prevalence), non-parametric tests (Mann–Whitney U) and Fisher’s Exact Test were utilized where appropriate to ensure the mathematical robustness of the comparisons. Associations between categorical variables were analyzed using the Chi-square test or Fisher’s exact test, as appropriate. To identify independent predictors of low BMD and to control for potential confounders (age, BMI, smoking status, ART duration, CD4+ nadir, and menopausal status), a multivariate logistic regression analysis was performed. Adjusted odds ratios (aOR) with 95% confidence intervals (95% CI) were calculated for each predictor. To facilitate clinical interpretation and meta-analytic utility, *p*-values were supplemented with effect size measures: Cohen’s d was calculated for continuous comparisons and Cramer’s V for categorical associations. Data management followed a strict complete case analysis protocol; only participants with full clinical and laboratory datasets were included to ensure data integrity. Furthermore, to mitigate the risk of Type I errors arising from multiple testing across tables, a cautious approach was adopted for the interpretation of *p*-values, particularly those near the significance threshold. A two-sided *p* < 0.05 was considered statistically significant.

### 4.6. Ethics and Institutional Review

This study was conducted in accordance with the Declaration of Helsinki and approved by the Ethics Committee of the “Dr. Victor Babes” Clinical Hospital for Infectious Diseases and Pneumophthisiology in Timisoara (approval no. 3082, 4 April 2023). Before enrollment, all participants received a detailed explanation of the research objectives, procedures, and potential risks. Participation was strictly voluntary and contingent upon obtaining written informed consent. Subject confidentiality and anonymity were maintained through rigorous data coding. Participants were explicitly informed of their right to withdraw from the study at any stage without consequence or impact on the quality of medical care provided.

## 5. Conclusions

The present study provides a comprehensive analysis of bone mineral density and body composition within a representative Romanian cohort of PLWH, revealing a high prevalence of skeletal demineralization affecting 58.3% of the participants at a significantly younger age that observed in the general population. These findings underscore that bone loss in this demographic is a multifaceted process driven by an intricate interplay of traditional risk factors, metabolic imbalances, and HIV-specific clinical markers.

The occurrence of osteoporosis at a mean age of only 45.7 years confirms a phenotype of accelerated skeletal aging. Consequently, we propose evidence-based screening criteria for the Romanian PLWH population, recommending the initiation of DXA and BIA assessments at the age of 40 for both sexes. Immediate screening is also warranted upon reaching clinical milestones such as incomplete immunological recovery (CD4+ < 500 cells/μL in women) or a history of TDF and PI use, irrespective of chronological age. A history of advanced HIV disease and low current CD4+ counts, particularly in our female subgroup (a trend requiring further large-scale validation), serve as potent predictors of bone mass depletion. The observation that lower immune recovery is intrinsically linked to skeletal integrity in women, suggests a distinct biological vulnerability. Furthermore, the strong correlation between smoking, hypertriglyceridemia, and reduced bone mineral density highlights the urgent need for integrated lifestyle interventions and aggressive management of dyslipidemia alongside antiretroviral therapy [70,71].

While the body mass index proved to be an inconsistent predictor of bone health, bioelectrical impedance analysis demonstrated high clinical utility in identifying subclinical bone mass loss, suggesting its role as a valuable, non-invasive adjunct to DXA for routine metabolic monitoring. The pervasive nature of Vitamin D deficiency and the high prevalence of cardiovascular and infectious comorbidities emphasize that skeletal health cannot be managed in isolation. In conclusion, the management of PLWH in the era of increased longevity must transition toward a multidisciplinary, holistic model where early screening (starting at age 40), aggressive correction of micronutrient deficiencies, and targeted smoking cessation programs are essential components to mitigate the long-term burden of fragility fractures and improve the quality of life in the aging HIV-positive population.

## Figures and Tables

**Table 1:** Baseline demographics and viro-immunological assessment.

	Total Cohort N = 180 (%)	Normal BMD N = 75 (%)	Osteopenia N = 87 (%)	Osteoporosis N = 18 (%)	*P* Normal BMD vs. Osteoporosis
**Demographics**					
**Sex**					
Female	62 (34.4)	27 (36.0)	30 (34.5)	5 (27.8)	0.452
Male	118 (65.6)	48 (64.0)	57 (65.5)	13 (72.2)	0.452
**Median age (years) ± SD**					
Overall	41.86 ± 12.69	39.86 ± 7.96	41.63 ± 9.90	45.77 ± 14.00	0.082
Female	41.44 ± 7.09	39.81 ± 4.13	42.10 ± 8.21	46.20 ± 11.77	0.065
Male	42.55 ± 12.86	39.89 ± 9.48	41.38 ± 10.73	45.61 ± 15.46	0.114
**Virological and immunological assessment**					
Median CD4+	525.89 ± 349.05	542.60 ± 377.95	513.26 ± 341.19	517.27 ± 264.63	0.710
Median CD4+—female	541.62 ± 377.06	590.07 ± 391.80	543.56 ± 376.66	268.40 ± 180.50	***<0.001***
Median CD4+—male	517.62 ± 334.78	515.89 ± 371.44	497.11 ± 323.35	613 ± 229.37	0.284
Viral load, median, copies/mL	335,277.68 ± 1,119,539.82	172,721.50 ± 627,014.37	555,718.65 ± 1,532,165.41	8250.00 ± 14,647.09	0.091
Undetectable viral load (<40 copies/mL), n (%)	128 (71.1)	54 (72.0)	63 (72.4)	11 (61.1)	0.354
ART duration, median, years ± SD	10.02 ± 7.00	9.50 ± 6.68	10.35 ± 7.45	10.23 ± 6.61	0.725
Time since HIV diagnosis, median, years	12.36 ± 8.70	12.01 ± 8.88	12.51 ± 8.76	12.69 ± 8.70	0.841

**Table 2:** Multivariate logistic regression analysis for predictors of low BMD.

Variable	Adjusted Odds Ratio (aOR)	95% Confidence Interval (CI)	*p*-Value
Age (years)	1.04	1.012–1.070	***0.007***
BMI (kg/m^2^)	1.458	0.421–5.047	0.314
Smoking Status (yes)	1.208	0.647–2.254	0.553
CD4+ count (current)	0.992	0.988–0.996	***0.003***
ART Duration	1.019	0.976–1.063	0.396
ART (number of regimens)	1.056	0.952–1.171	0.304

**Table 3:** Comparative Analysis of Demographic, Clinical, and Metabolic Characteristics Stratified by BMD Status in PLWH.

	Total Cohort N = 180 (%)	Normal BMD N = 75 (41.7)	Osteopenia N = 87 (48.3)	Osteoporosis N = 18 (10)	*P* Normal BMD vs. Osteoporosis
**Demographics**					
**Sex**					
Female	62 (34.4)	27 (36.0)	30 (34.4)	5 (27.8)	0.512
Male	118 (65.6)	48 (64.0)	57 (65.6)	13 (72.2)	0.512
**Age groups (years)**					
20–30 years	18 (10.0)	5 (6.7)	11 (12.6)	2 (11.1)	0.482
31–40 years	88 (48.9)	43 (57.3)	38 (43.7)	7 (38.9)	0.154
41–50 years	43 (23.9)	19 (25.3)	21 (24.1)	3 (16.7)	0.621
51–60 years	26 (14.4)	8 (10.7)	14 (16.1)	4 (22.3)	0.234
61–70 years	3 (1.7)	-	2 (2.3)	1 (5.5)	0.081
71–80 years	2 (1.1)	-	1 (1.2)	1 (5.5)	0.062
**HIV-Related Factors**					
**Clinical Stage**					
HIV infection	51 (28.3)	20 (26.7)	26 (29.9)	5 (27.8)	0.842
AIDS	129 (71.7)	55 (73.3)	61 (70.1)	13 (72.2)	0.842
**Lifestyle and Habits**					
Smoking	105 (58.3)	44 (58.7)	47 (54.0)	14 (77.8)	***<0.001***
Alcohol Consumptions	24 (13.3)	10 (13.3)	11 (12.6)	3 (16.7)	0.715
Sedentary Lifestyle	85 (47.2)	33 (44.0)	45 (51.7)	7 (38.9)	0.354
**Nutritional Status**					
**BMI Classification**					
Underweight	28 (15.6)	8 (10.7)	15 (17.2)	5 (27.8)	0.092
Overweight	42 (23.3)	16 (21.3)	21 (28.0)	5 (27.8)	0.641
Obesity	21 (11.6)	9 (12.0)	11 (12.6)	1 (5.5)	0.428
**BIA Parameters**					
High Visceral Fat	29 (16.1)	7 (9.3)	18 (20.7)	4 (22.3)	0.118
Low Total Bone Mass	93 (51.7)	38 (50.7)	45 (51.7)	10 (55.6)	***<0.001***
**Lipid Profile**					
Hypercholesterolemia	49 (27.2)	21 (28.0)	22 (25.9)	6 (33.3)	0.684
High LDL-C	139 (77.2)	65 (86.7)	69 (79.3)	5 (27.8)	0.075
High HDL-C	18 (10.0)	8 (10.7)	10 (11.5)	-	0.214
Hypertriglyceridemia	48 (26.7)	14 (18.7)	26 (29.9)	8 (44.4)	***<0.001***
**Biomarkers**					
Calcium Deficiency	19 (10.6)	7 (9.3)	10 (11.5)	2 (11.1)	0.842
Magnesium Deficiency	6 (3.3)	2 (2.7)	3 (3.4)	1 (5.5)	0.621
Phosphorus Deficiency	16 (8.9)	3 (4.0)	10 (11.5)	3 (16.7)	0.094
Vitamin D Deficiency	134 (74.4)	55 (73.3)	66 (75.9)	13 (72.2)	0.815

BMD = Bone Mineral Density; HIV = Human Immunodeficiency Virus; AIDS = acquired immunodeficiency syndrome; LDL-C = low-density lipoprotein cholesterol; HDL-C = high-density lipoprotein cholesterol.

## Data Availability

The available data can be provided upon request to the corresponding author (due to internal regulations of the hospital—Regulation UE nr. 679 from 2016 regarding protection of personal data).

## Abbreviations

The following abbreviations are used in this manuscript:

AIDS	Acquired Immunodeficiency Syndrome
ART	antiretroviral therapy
ARV	antiretroviral
BIA	bioelectrical impedance analysis
BMD	Bone Mineral Density
BMI	body mass index
CD4	Cluster of Differentiation 4
CDC	Centers for Diseases Control and Prevention
DXA	dual-energy X-ray absorptiometry
eGFR	Estimated Glomerular Filtration Rate
HBV	Chronic Hepatitis B
HCV	Chronic Hepatitis C
HDL-C	high-density lipoprotein cholesterol
HIV	Human Immunodeficiency Virus
HIV-RNA	Human Immunodeficiency Virus Ribonucleic Acid
IQR	interquartile range
LDL-C	low-density lipoprotein cholesterol
MSM	men who have sex with men
MTRs	multi-tablet regimens
PLWH	people living with HIV
RANKL	Receptor Activator of Nuclear Factor Kappa-B Ligand
SD	Standard Deviation
STRs	Single-Tablet Regimens
TB	Tuberculosis
TC	total cholesterol
TDF	tenofovir disoproxil fumarate
TGs	triglycerides
WHO	World Health Organization
